# Optical Fiber Ball Resonator Sensor Spectral Interrogation through Undersampled KLT: Application to Refractive Index Sensing and Cancer Biomarker Biosensing

**DOI:** 10.3390/s21206721

**Published:** 2021-10-10

**Authors:** Daniele Tosi, Zhannat Ashikbayeva, Aliya Bekmurzayeva, Zhuldyz Myrkhiyeva, Aida Rakhimbekova, Takhmina Ayupova, Madina Shaimerdenova

**Affiliations:** 1 School of Engineering and Digital Sciences, Nazarbayev University, Nur-Sultan 010000, Kazakhstan; zhashikbayeva@nu.edu.kz (Z.A.); abekmurzayeva@nu.edu.kz (A.B.); zhuldyz.myrkhiyeva@nu.edu.kz (Z.M.); aida.belova@nu.edu.kz (A.R.); takhmina.ayupova@nu.edu.kz (T.A.); madina.shaimerdenova@nu.edu.kz (M.S.); 2 Laboratory of Biosensors and Bioinstruments, National Laboratory Astana, Nur-Sultan 010000, Kazakhstan

**Keywords:** optical fiber biosensor, optical fiber sensor, ball resonator, optical fiber spherical tip, digital signal processing, Karhunen−Loeve transform (KLT), cancer biomarker diagnostic

## Abstract

Optical fiber ball resonators based on single-mode fibers in the infrared range are an emerging technology for refractive index sensing and biosensing. These devices are easy and rapid to fabricate using a CO_2_ laser splicer and yield a very low finesse reflection spectrum with a quasi-random pattern. In addition, they can be functionalized for biosensing by using a thin-film sputtering method. A common problem of this type of device is that the spectral response is substantially unknown, and poorly correlated with the size and shape of the spherical device. In this work, we propose a detection method based on Karhunen−Loeve transform (KLT), applied to the undersampled spectrum measured by an optical backscatter reflectometer. We show that this method correctly detects the response of the ball resonator in any working condition, without prior knowledge of the sensor under interrogation. First, this method for refractive index sensing of a gold-coated resonator is applied, showing 1594 RIU^−1^ sensitivity; then, this concept is extended to a biofunctionalized ball resonator, detecting CD44 cancer biomarker concentration with a picomolar-level limit of detection (19.7 pM) and high specificity (30–41%).

## 1. Introduction

Optical fiber biosensors make use of optical fiber waveguides for detecting biological analytes on the fiber surface, using suitable biofunctionalization methods that allow sensors to have excellent sensitivity and high specificity [1], and find important applications as immunosensors [2] in the diagnostic of cancer [2,3], cardiac [4], and urologic biomarkers [5], as well as in modern wearable devices [6]. Biosensors base their working principle on refractive index (RI) detection [7]; using a suitable functionalization method, it is possible to convert a refractometer into a biosensor and measure the response to a specific analyte as well as the specificity of detection.

An important set of optical fiber biosensors is based on single-mode fibers (SMFs) operating in the infrared range. The use of SMF fibers, in lieu of large-core or microstructured fibers or plastic fibers, yields a great simplicity in the fabrication and interrogation of the devices using telecom grade equipment [8]. Since SMF fibers have been widely standardized in terms of materials, geometrical size, and type of coatings (such as ITU-T G.652.D standard), it is possible to manufacture devices using the same equipment and components used in optical communications (for example, fiber splitters, lasers and LED sources, photodiodes, filters). In return, SMF-based devices working at infrared wavelengths have exceptionally low fiber losses that enable remote sensing, great accuracy which results in a low limit of detection and high figures of merit and have a great availability of splicing and interconnection tools.

The working principle of most fiber optic biosensors relies on the detection of the reflection or transmission spectrum of the sensor [7]. Within the spectrum, we can identify and monitor specific spectral features, that depend on the RI. The shape of the spectral envelope we can observe on the analyzer is directly tied to the type of sensor and is often referred to as a spectral “signature” or “fingerprint”. In each spectral signature, we can identify one or more features, such as reflection peaks or transmission dips, and track their wavelength or intensity to obtain an estimate of the RI change.

Fiber Bragg gratings (FBGs) represent the widest approach for RI sensing and biosensing using SMF fibers in the infrared range [9]. While a standard FBG is not RI sensitive, we can modify the geometry of the grating or the shape of the fiber to have highly sensitive biosensors. The most common approach is to use an etched FBG which behaves as a narrowband notch filter with a sensitivity depending on the fiber thickness [10]. Another important approach makes use of a tilted FBG, which encodes the RI into cladding modes propagated out of the fiber [11]; tilted gratings can also increase their sensitivity by etching the fiber waveguide [12] or using plasmonic effects generated in a thin metallic film [13]. Long-period gratings (LPG), on the other hand, are gratings having an extended period, and working as band-reject filters combining co-propagating fiber modes [14].

Interferometers are also a common type of RI sensors and biosensors, making use of structures that allow waves exposed to the external RI to interfere with different optical path differentials [15] Fabry−Perot interferometers fabricated with SMFs use different fiber spans as mirrors, usually encapsulating an air gap [16]; structures based on a metallic mirror for high reflectivity [17] as well as low-finesse all-glass interferometers [18] have been reported.

The spectral fingerprints of gratings and interferometers have high fringe visibility and allow simple detection using signal processing methods common in data acquisition and analysis. FBGs have a sharp reflection peak with reflectivity that is tens of dB higher than the noise floor, and the RI is detected by measuring the wavelength shift. For tilted FBGs, the RI is encoded in the cladding modes, which are visualized as a spectral comb; the spectral features under investigation are usually the cut-off mode [19], very common in the sensing of large RI changes, or the amplitude of a resonant mode, which is more suitable for plasmonic gratings [13]. LPGs have a much wider spectrum, encompassing hundreds of nanometers of bandwidth, and a wavelength shift is observed as the RI increases [7]. Conversely, interferometers have a spectrum with a periodic pattern, with a fringe visibility that depends on the finesse of the cavity; the detection is performed by either measuring a spectral peak or dip, or performing the differential between multiple spectral features, such as in the case of dual RI/temperature sensors [4].

Recently, the use of fiber-optic ball resonators has been introduced [20,21]. This type of sensor operates on the same SMF fiber and infrared wavelength range as gratings and interferometers, but has a much faster and easier fabrication process, as we can reuse the same process developed for the manufacturing of diffractive tip lenses for microscopy [22]. Using a CO_2_ laser splicer, this process has a high throughput and is implemented as a single step with a few seconds duration.

Ball resonators do not have a well-defined spectrum, and are rather low-reflectivity devices with fringe visibility in the order of ~1% or below; most importantly, it appears that each ball resonator is characterized by a different spectral fingerprint that is unique for the device and appears as a quasi-random reflection spectrum. Hence, unlike gratings or interferometers that have a well-defined spectrum that entirely depends on the parameters of the device (such as the Bragg wavelength for an FBG, or the cavity length for a Fabry−Perot interferometer), a ball resonator has a spectral fingerprint that we can approximate as a random signal, and changes in wavelength and amplitude in a similar fashion to the other RI sensors and biosensors.

The works presented so far propose a demodulation method for ball resonators that is based on feature extraction, by measuring the most significant spectral feature and tracking its amplitude [20] or pitch wavelength [21]. This method has been demonstrated both by analyzing the return loss [20], or each individual polarization for an enhanced sensitivity [23]. Feature extraction is, however, a sub-optimal method as it requires knowledge of the spectral response of the sensor, which cannot be achieved before the fabrication. A more ideal scenario for the detection of a ball resonator would rely on a method for demodulation that processes the reflection spectrum without having a predefined spectral feature, but can rather work with any spectral fingerprint.

In this work, we introduce a new demodulation method for ball resonators that is based on the KLT (Karhunen−Loeve transform) [24] of the undersampled ball resonator spectrum. We show that this simple yet effective method can return an output that depends on the RI, and we show that we can use this method for RI sensing; subsequently, we extend this method to a typical case scenario of a fiber-optic biosensor, by detecting various concentrations of CD44 proteins. The increased CD44 protein level in serum can be an indicator of tumor burden and metastasis in several types of cancer, such as breast, gastric, and colon, and hence it can be used as a biomarker [25,26]. We show that the KLT can work regardless of the shape of the spectral fingerprint.

The paper is arranged as follows: Section 2 describes the fabrication process, the biofunctionalization, and the interrogation setup for the sensors used in this work; Section 3 shows the principle of operation of the KLT applied to the ball resonator detection; Section 4 shows the results of the work, by first analyzing the performances of the KLT and then showing the evidence of RI sensing and CD44 biosensing case scenarios; Section 5 briefly discusses the impact of KLT in sensor detection; finally, Section 6 draws the conclusions.

## 2. Materials and Methods

### 2.1. Fabrication of the Ball Resonator Sensor

A sensor based on optical fiber with a spherical tip at the end-point was fabricated on a standard single-mode fiber (SMF-28) using a CO_2_ laser splicer (Fujikura LZM-100). This was done by the following process: aligning two fibers, splicing, and then subjecting the produced structure to high laser power which is specific to the fabrication equipment. As a result, a spherical tip at the end of the fiber was formed while undergoing breaking close to the splicing point. Fabrication parameters are shown in Table S1.

**Figure 1:**
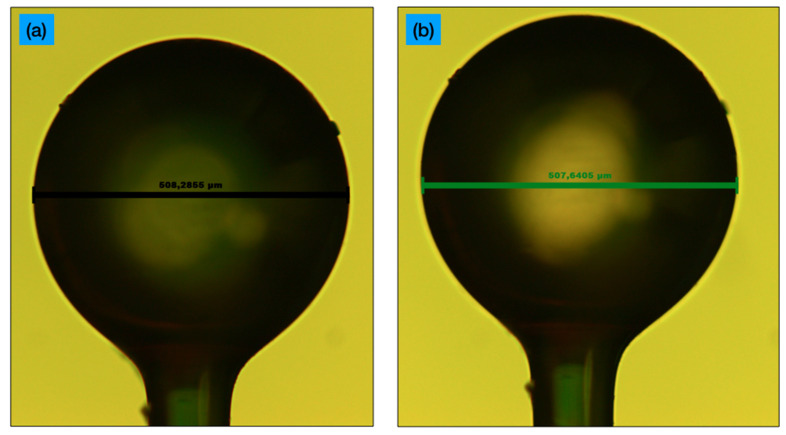
Photograph of the fabricated ball resonator under an optical microscope; (**a**) cross-section along the horizontal plane; (**b**) vertical plane.

The photograph of the sensor fabricated and subsequently biofunctionalized for CD44 detection is shown in Figure 1. The cross-sections show that the diameters are 508.2 μm along the horizontal direction, and 507.6 μm along the vertical direction; the estimated eccentricity is equal to 0.049.

### 2.2. Experimental Setup

**Figure 2:**
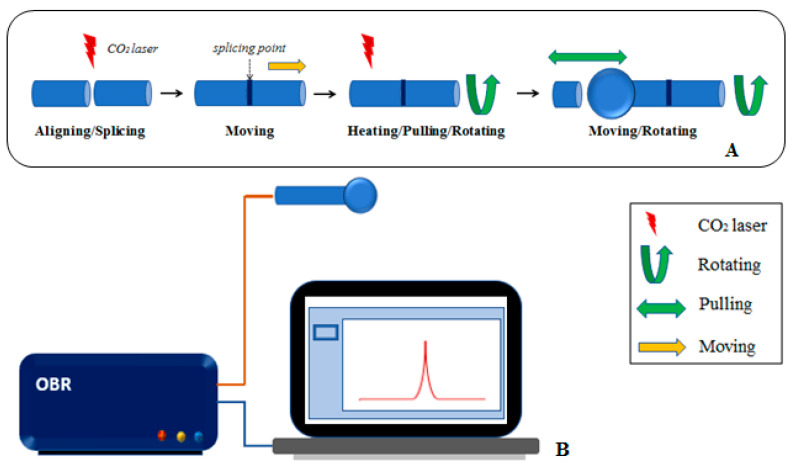
Experimental set-up used in this study; (**A**) fabrication of ball resonator sensor on a CO_2_ splicing machine; (**B**) interrogation of the sensor with optical backscatter reflectometer.

The experimental setup used in the whole process of interrogation of ball resonators is shown in Figure 2.

The interrogator is an optical backscatter reflectometer (OBR; OBR4600, Luna Inc., Roanoke, VA, USA), with the following parameters: scan range 1525–1610.6 nm; gain 0 dB; resolution bandwidth 0.257 GHz.

Spectra were filtered with a low-pass filter: Chebyvhev type-I filter, 7th order, 0.0084 normalized cut-off frequency. After filtering, and removing the transient of the filter, the analysis was carried out on 40,000 samples, wavelength range 1528.7–1580.1 nm.

All evaluations of KLT performance figures and computation times were implemented in MatLab (Mathworks, Natick, MA, USA), running on a MacBook Pro 2.6 GHz, 16 GB RAM.

### 2.3. Refractive Index Calibration

Refractive index (RI) calibration was done by measuring the change in spectra to RI change using solutions with different RI values. Several concentrations of sucrose in water (w/v %) starting from 10% to 13.53% in 9 increments of ~0.49% corresponding to RI values from 1.3478 to 1.3539 for a total change of 6.1 × 10^−3^ RIU in steps of 5.86 × 10^−4^.

### 2.4. CD44 Functionalization, Measurement, and Specificity

The surface of the ball resonator underwent cleaning with Piranha solution (4:1 = H_2_SO_4_:H_2_O_2_) followed by silanization with 1% aminopropyltrimethoxysilane (APTMS) utilized to obtain amine groups on its surface for the further attachment with the gold. The gold sputtering machine (Q150T Plus, Quorum Technologies Ltd., Lewes, UK) allowed coating the surface of the ball resonator with the gold at a thickness of 30 nm followed by annealing at high temperature (200 °C) for 2 h. Furthermore, the gold-coated sensor was pretreated with 95% 11-Mercaptoundecanoic acid (MUA) for 16 h at 2–4 °C and activated with EDC/NHS (1-ethyl-3-carbodiimide hydrochloride/N-Hydroxysuccinimide) for 15 min. Finally, the ball resonator was incubated on a shaker for 30 min with 8 mg/mL anti-CD44 antibody and blocked with 1% BSA.

The sensor functionalized with CD44 antibody was employed further for the CD44 protein detection. Optical backscattering reflectometer (OBR) was utilized for the signal detection over the CD44 protein measurement analysis at the concentration range from 0.006 nM to 100 nM. For detection purposes, the functionalized ball resonator was immersed in the vial containing the 200 µL CD44 protein solution.

To conduct the specificity analysis the IL-4 protein measurement at the same concentration range was utilized as a control. To validate the performance of the obtained ball resonators, the experiments were conducted at the same conditions and parameters.

## 3. Interrogation of Ball Resonators with KLT

The reflection spectrum *R_Ref_*(*λ*) of a ball resonator, where *λ* is the wavelength in the infrared range, can be approximated in reference conditions as [20]:

(1) $$R_{Ref}\left(\lambda \right)=c\cdot s\left(\lambda \right),$$

where *c* is a constant, that defines the baseline level of the reflectivity, and *s*(*λ*) is the spectral signature of the ball resonator, which appears as a fluctuating spectral envelope having a shape different for each sensor. When the RI changes, the reflection spectrum changes in amplitude, by the effect of the variation of reflectivity at the interface between the glass fiber sphere and the outer medium, and experiences a slight wavelength shift as a consequence of the phase change of the reflection coefficient at each reflection. The measured spectrum *R_Meas_*(*λ*), therefore, can be approximated as:

(2) $$R_{Meas}\left(\lambda \right)=\left(c+\Delta c\right)\cdot s\left(\lambda -\Delta \lambda \right),$$

where Δ*c* is a term that shows the change of spectral amplitude, and Δ*λ* is a wavelength shift. In the case of a small change of RI, in the order of 10^−3^ or below, as shown in [20], we can approximate the amplitude and wavelength shifts as linear terms:

(3) $$R_{Meas}\left(\lambda \right)\approx \left(c+k_{a}\Delta n\right)\cdot s\left(\lambda -k_{w}\Delta n\right),$$

where the terms *k_a_* and *k_w_* represent the amplitude sensitivity, expressed usually in dB/RIU, and the wavelength sensitivity, typically in nm/RIU, respectively. As shown in [20,23], amplitude and wavelength sensitivity vary in each sensor and depend on the shape of the resonator.

The Karhunen−Loeve transform (KLT), implemented as in Maccone’s algorithm [24,27,28] is then applied on each measured spectrum, treated as a noisy signal. The approach described by Maccone is to decompose the measured spectrum *R_Meas_*(*λ*) into a set of orthonormal bases:

(4) $$R_{Meas}\left(\lambda \right)=\sum_{n=1}^{\infty }Z_{n}\cdot \phi_{n}\left(\lambda \right),$$

where the coefficients *Z_n_* represent the “magnitude” of each *n*-th basis, and the terms *ϕ_n_*(*λ*) are orthonormal bases. The orthonormality condition, computed between the minimum (*λ_min_*) and maximum (*λ_min_*) wavelength values of the optical interrogator, reads as:

(5) $$\int_{\lambda_{min}}^{\lambda_{max}}\phi_{n}\left(\lambda \right)\phi_{m}\left(\lambda \right)d\lambda =\delta_{mn},$$

with *δ_mn_* being the Kronecker delta.

The difference between the KLT and other transform methods is that while in classical algorithms for signal processing the set of basis *ϕ_n_* is predefined, in the KLT they are entirely dependent on the signal under analysis. For example, in the Fourier transform the set of orthonormal basis is made of sine functions of different frequency; in wavelet transforms the set of bases are wavelet functions having different scale and pitch.

However, since the spectra of the ball resonators are not inferred prior to their fabrications, since there is a very poor correlation between the fabrication parameters and the reflection spectrum as clearly observed in previous works [20], the KLT offers a superior method for spectral processing since it can form a set of orthonormal basis that is entirely data-dependent. As a consequence, the coefficients *Z_n_* are random variables that measure the magnitude of each basis [29].

To resolve the KLT, we need to find the statistical bases *ϕ_n_*(*λ*); Maccone demonstrated in his work [28] that the set of bases *ϕ_n_*(*λ*) is the eigenfunction of the autocorrelation of the signal *R_Meas_*(*λ*). The problem of finding the eigenfunctions is integral, and since we can treat the spectral signatures as quasi-random signals, rather than analytical functions, we cannot have a closed-form solution. Maccone demonstrated this method to estimate the frequency of a sine function buried into noise [24], but this problem is different from the one under analysis as, in the case of ball resonator spectra, we need to measure the amplitude and wavelength shifts of an almost flat spectrum with extremely low fringes.

However, from an experimental point of view, we do not work with continuous signals, but rather the OBR interrogator provides a digitally sampled signal. In this case, we can translate the continuous-time processing into a discrete version of the KLT, which is much faster to implement.

We consider the measured spectrum as *S*[*m*] where *m* = 1, 2, …, *M* is the sample number of a digital signal. Whereas in the continuous KLT we need to identify the eigenfunctions of the autocorrelation, in the digital domain the KLT aims at finding the eigenvalues of the autocorrelation matrix. Hence, in other words, the bases *ϕ_n_* become the eigenvectors, and the coefficients *Z_n_* become the eigenvalues of the autocorrelation matrix.

Computationally, we first apply to compute the autocorrelation matrix ***R_SS_*** of the spectrum:

(6) $$R_{SS}=S\left[m\right]S^{T}\left[m\right]=\;\left[\begin{array}{cc} \begin{array}{cc} S\left[1\right]S\left[1\right] & S\left[1\right]S\left[2\right]\\ S\left[2\right]S\left[1\right] & S\left[2\right]S\left[2\right] \end{array} & \begin{array}{cc} \cdots & S\left[1\right]S\left[m\right]\\ \cdots & S\left[1\right]S\left[m-1\right] \end{array}\\ \begin{array}{cc} \vdots & \vdots \\ S\left[m\right]S\left[1\right] & S\left[m-1\right]S\left[2\right] \end{array} & \begin{array}{cc} \ddots & \vdots \\ \ldots & S\left[m\right]S\left[m\right] \end{array} \end{array}\right],$$

where the operator *T* stands for transpose. We note that, from the notation of Equation (6), we omitted the expected value operator, since in our experimental arrangement we do not have a possibility to estimate the expected values of *S*[*m*] but rather we have a single realization of the spectral measurement, and we generate the autocorrelation matrix from this signal alone.

To estimate the eigenvalues and eigenvectors, we can apply the singular value decomposition (SVD) [30]:

(7) $$R_{SS}=VDV^{-1},$$

in which: ***D*** is a diagonal matrix that has all eigenvalues on the main diagonal; ***V*** is the eigenvectors matrix, that has on each *i*-th column the eigenvector corresponding to the *i*-th eigenvalue, i.e., the ***D_ii_*** element of ***D***. Since ***R_SS_*** is a symmetric matrix, the eigenvalues are all real. We label the eigenvalue string as *ξ* = [*ξ*_1_, *ξ*_2_, …, *ξ_M_*], such that *ξ_i_* is the *i*-th element of the diagonal of ***D***.

Through implementing the SVD, we can sort the matrices ***D*** and ***V*** such that the eigenvalues are sorted in ascending order:

(8) $$\xi_{M}\geq \xi_{M-1}\geq \ldots \geq \xi_{1}.$$

The SVD is the key step of the KLT because the eigendecomposition allows the identification of one eigenvalue having a magnitude much larger than all the other ones. In this sense, the largest eigenvalue *ξ_M_* represents the main outcome of the KLT.

The main problem in solving Equation (7) is computational, as ***R_SS_*** is a *M* x *M* matrix, and spectral acquisition on the OBR is performed with *M* = 65,536 samples. The eigendecomposition process implies solving an M-size linear system of equations, which is a ~O(M^3^) process. Hence, we adjust the original KLT algorithm by undersampling the acquired reflection spectra, reducing the complexity; this can keep the computation time below 1 s while guaranteeing a reliable output.

In formulas, we downsample the spectrum by a rate *K*, obtaining a new signal *T*[*p*] with length *P* = *M*/*K*:

(9) T[p]=T[1,2,…,P]=[S[1],S[K+1],S[2K+1],…,S[(P−1)K+1]

and then repeat Equations (6) and (7) to the signal *T*, rather than on the original spectrum *S*, by first building the *P* × *P* autocorrelation matrix and then determining the *P*-size eigenvector string. In this case, the main eigenvalue is *ξ_P_*.

The undersampled KLT accomplishes two tasks: it allows the analysis of the whole spectrum of the ball resonators, hence having a much better chance to build the eigenbasis that show the maximum sensitivity to the RI change; at the same time, it keeps the computational complexity low, allowing fast estimation of the KLT output.

As shown by Maccone [28], for an M-size KLT calculation, the value of the main eigenvalue *ξ_M_* grows linearly:

(10) $$\frac{\partial \xi_{M}}{\partial M}=const.$$

Therefore, we can normalize the main eigenvalue by the length of the analyzed signal, to obtain an output that is independent of the sampling rate (which can therefore allow comparison of the spectra acquired with a different number of samples). Here, we label as *ω* the output of the KLT, defined as:

(11) $$\omega =\frac{\xi_{P}}{P}=\frac{K}{M}\xi_{P}.$$

**Figure 3:**
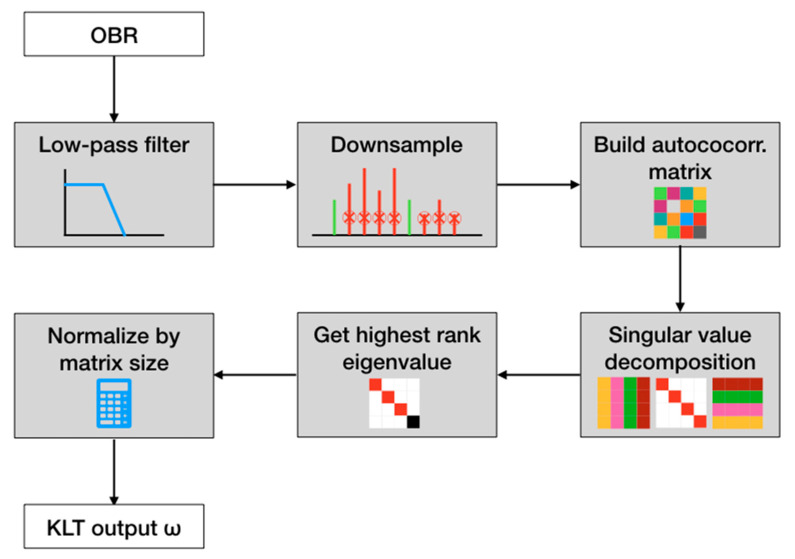
A flow chart of the undersampled KLT is used to demodulate the spectrum of the ball resonators.

To summarize, the undersampled KLT method works as follows (and also as shown in Figure 3):

- Acquire the reflection spectrum of the ball resonator;
- Low-pass filter to clean the higher frequency noise, and obtain the signal *S*[*m*] with *M* = 40,000 samples.
- Downsample *S*[*m*] by a rate *K*, obtaining the new signal *T*[*p*] with size *P* = *M*/*K* samples;
- Build the *P* × *P* autocorrelation matrix of *T*[*p*];
- Apply the SVD to the autocorrelation matrix, and identify the eigenvalue string;
- Compute the KLT output *ω* as the highest rank eigenvalue normalized by *P*.

## 4. Results

### 4.1. Operation and Performances of the KLT

In this section, we analyze the typical output of the KLT, and how the performances are affected by the main parameters. We analyze the sensor described in Section 2, exposed to a reference RI value, as a benchmark.

**Figure 4:**
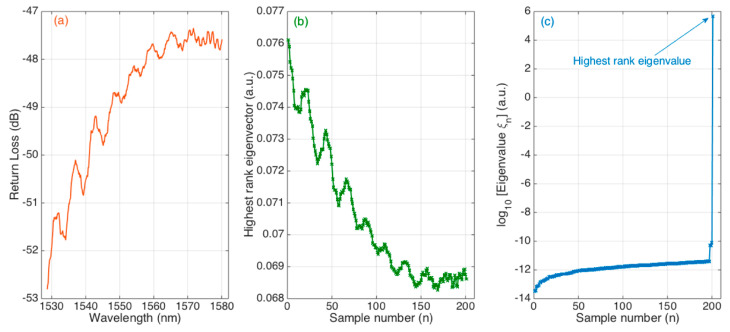
Overview of the operation of the undersampled KLT applied to the spectrum of a ball resonator. (**a**) The reflection spectrum of the ball resonator, acquired by the OBR and processed. (**b**) The eigenvector corresponding to the highest rank eigenvalue, acquired over 201 samples. (**c**) Eigenvalue string, reporting the magnitude (in logarithmic units) of all 201 eigenvalues sorted in ascending order.

Figure 4 shows the operation of the KLT, with *K* = 200 downsampling rate (201 samples under analysis). The first chart shows the reflection spectrum of the ball resonator, which appears in analogy with previous works [20] and shows an almost random pattern of shallow peaks, with amplitude <1 dB.

By applying the KLT, we can obtain the eigenvalues and eigenvectors of the autocorrelation matrix of the downsampled signal. The eigenvector corresponding to the maximum eigenvalue is shown in the second chart. We see that the envelope resembles the flipped spectrum of the ball resonator, which shows that the SVD operation can single out the main eigenbasis of the ball resonator spectrum.

In Figure 4c, we show the eigenvalue string, with all values ordered in ascending order; for better visibility, we display the data in logarithmic units. We can see that the main eigenvalue stands out from the other values: the highest rank eigenvalue is equal to 4.8 × 10^5^, while the second eigenvalue 7.7 × 10^−11^. This means that the KLT entirely encodes the ball resonator spectrum in the highest rank eigenvalue, whereas all the other eigenvalues are 15 orders of magnitude below this value.

This observation was reported by Maccone in the problem of extracting a signal buried in noise [24]; in this work, as noise is not very dominant in the optical spectrum, we have a wider divergence between the highest eigenvalue and the other ones. In the case of a spectrum with more noise (for example, by omitting the low-pass filter), we would also observe a rise of the lower-rank eigenvalue, while the main one would maintain a similar value and shape of its eigenvector.

The performance rates of the KLT depend on the choice of undersampling rate *K*: by choosing a higher value of *K,* the KLT is faster, as the SVD operates on a matrix with a size proportional to ~1/*K*. On the other hand, by working with severely undersampled spectral signals, we lose spectral features that can become part of the KLT eigenbasis.

**Figure 5:**
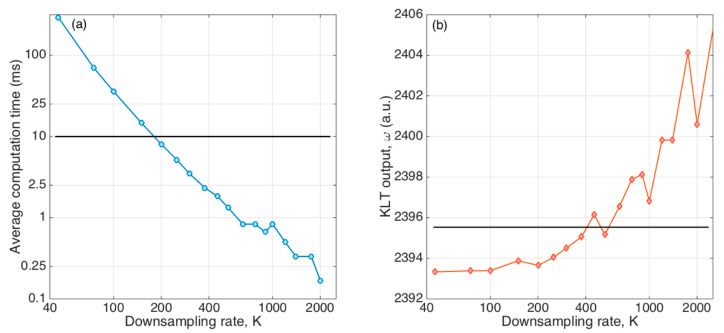
Performance analysis of the KLT as a function of the undersampling rate *K*. (**a**) Average computation time over 100 KLT calculations as a function of *K*; the horizontal line shows the limit of 10 ms computation time for rapid computing. (**b**) KLT output *ω* as a function of *K*; the horizontal line shows the limit of 0.1% variation of *ω* from the reference value computed with *K* = 45.

We can evaluate experimentally the range of *K* values that optimizes the KLT; this process is shown in Figure 5. The left chart shows the computation time, averaged over 100 different KLT calculations, for *K* values ranging from 45 (*P* = 889) to 2000 (*P* = 21). We can see that the computation time increased significantly for lower values of *K*, following an almost linear pattern when data are displayed in logarithmic units. A good selection was obtained by imposing a fast computation, which would not slow down the acquisition rate of the OBR. Considering that the OBR works at ~1 Hz on the continuous acquisition on the full bandwidth, by imposing a computation time ≤10 ms we guaranteed that the data processing time was <1% of the acquisition sampling time; this value corresponds to *K* = 172, and therefore a choice of *K* > 172 satisfies the fast computation criteria. Considering that the data processing was implemented on a capable processor, this criterion would also guarantee a rapid computation in most experimental conditions. By imposing, instead, a looser constraint on the acquisition time as ≤100 ms, we obtained *K* > 66.

The second chart shows how the KLT output *ω*, computed on the same dataset, changed as a function of *K*. We have seen in Equation (10) that the output of the KLT should be constant with respect to the size of the autocorrelation matrix; a good evaluation of the quality of the KLT is to evaluate the accuracy of this statement, and therefore evaluate the change of *ω* from the reference level (hereby obtained with the lowest value *K* = 45) as *K* increases. At first, we notice that in all conditions the variation of *ω* was limited to 0.46% change from 2393.3 to 2404.1, and therefore in all conditions, even working on very small matrices, we can guarantee that the KLT has good effectiveness. Empirically, we can define it as a good choice of *K,* a value that limits the deviation of *ω* to ≤0.1% from the reference value; this corresponds to *K* < 393.

Hence, a good choice according to this experimental analysis is 172 < *K* < 393; in the following analysis, we always set *K* = 200.

### 4.2. Refractive Index Sensitivity

**Figure 6:**
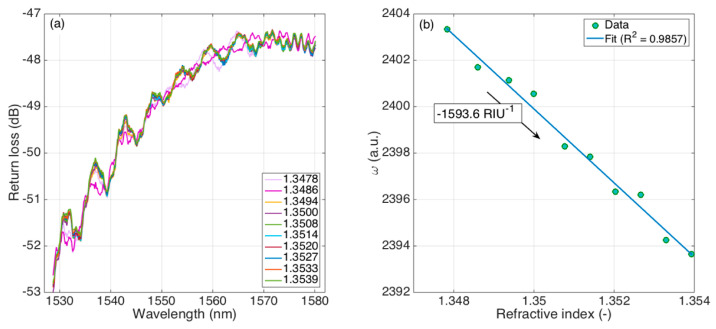
Refractive index sensitivity of the ball resonator interrogated with the KLT method. (**a**) Reflection spectra of the ball resonators, for different RI values ranging from 1.3478 to 1.3539. (**b**) KLT output *ω* as a function of the RI; the linear fit shows the sensitivity as −1593.6 RIU^−1^.

The refractive index calibration of the ball resonator is shown in Figure 6. The first chart shows the reflection spectrum of the ball resonator, for RI values ranging from 1.3478 to 1.3539 (for a total change of 6.1 × 10^−3^ RIU (refractive index units). We can see that the spectral changes combined, in agreement with Equations (2) and (3), a wavelength shift (observed in the proximity of the shallow spectral fringes) and an intensity change, which is observed nearby the spectral peaks. Overall, by tracking each spectral feature alone, we cannot define an easy interrogation method.

The KLT, as shown on the right chart, was, however, effective in returning an output that had a clear RI dependence, despite the lack of clear spectral features. The KLT output *ω* changed linearly as the RI increased, following a path similar to other fiber-optic refractometers interrogated on a narrow RI range [7].

The sensitivity can be estimated by applying a linear fit to the *ω* vs. RI calibration function; the resulting sensitivity was −1593.6 RIU^−1^, obtained with the coefficient of determination R^2^ = 0.9857 which showed an excellent agreement between the *ω* decrease and the increase of RI. This corresponds to a decrease of *ω* equal to 0.066% per each RI increase of 10^−3^ RIU.

### 4.3. Biosensing Capability: Detection of CD44

**Figure 7:**
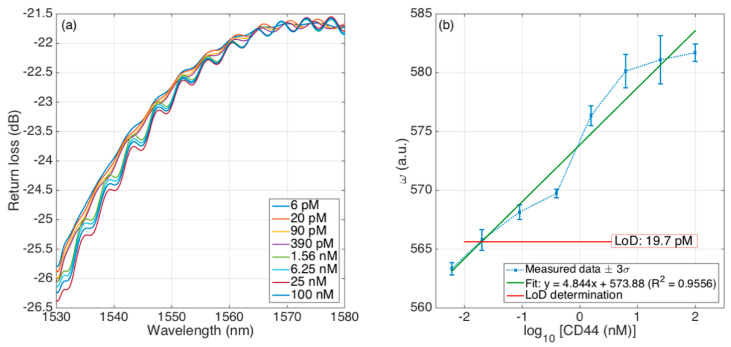
Detection of CD44 protein with a biofunctionalized gold-coated ball resonator. (**a**) The reflection spectrum of the ball resonator for different CD44 concentrations, ranging from 6 pM to 100 nM; (**b**) KLT response for each concentration, reporting the standard deviation of data acquired over 10 min, the log−linear fit, and the LoD determination.

Figure 7 shows the response of the functionalized ball resonator to the CD44 protein, with concentrations ranging from 6 pM to 100 nM. The first chart shows the spectral response of the sensor. In comparison to Figure 6, we observe that the spectrum maintained a similar envelope with a smoother pattern near the peaks, but with a much higher reflectivity value due to the gold-coating process, which increases the wall reflectivity at the interface between the spherical tip and the outer medium. Once again, we observe that the detection was hampered by the uneven shape of the spectral fingerprint, which prevented a simple feature extraction algorithm to work.

By processing the spectral envelope with the undersampled KLT, on the other hand, we obtained a much clearer response; this is shown in Figure 7b, where we report the change of *ω* as related to the CD44 concentration change. Hereby, the datapoint represents the average of the sensor response over 10 min of acquisition, while the error bars report ±3 times the standard deviation, as commonly performed for fiber optic biosensors [1].

We observed that the baseline value of *ω* changed from the previous RI calibration; this is due to the different spectral shape, and particularly the different change of spectrum observed over the acquisition window; for the RI calibration this change was 5.2 dB, while after biofunctionalization (and particularly, after gold coating) the change was limited to 4.0 dB. However, once again we have a clear pattern that increases with the concentration of CD44. The standard deviation appears to be lower at the lowest concentrations, while it tends to increase at higher concentrations reaching its maximum value at 25 nM.

The quality of the detection is shown by a log−linear fit, that relates the increase of *ω* to a 10× increase of CD44 concentration; the fit shows that *ω* increases by 4.844 for each 10× concentration increase, with an R^2^ parameter equal to 0.9556 which shows a good match between experimental data and the fit.

We can also infer the limit of detection (LoD) of this sensor, by using the method reported by Chiavaioli et al. [7]: considering as blank level the lowest concentration (6 pM) and adding three times the worst-case standard deviation, we can obtain the level of *ω* that defines the LoD (indicated in red in Figure 7b): this level corresponds to 19.7 pM, which shows that the proposed method based on KLT can successfully demodulate the spectral fingerprint with shallow and uneven patterns into a biosensor with a clear response, and LoD at picomolar-level concentration.

The specificity of the sensor has been evaluated by comparing the response to CD44 to the response to a control (IL4). Specificity tests have been performed by analyzing the response to CD44 at four different concentrations (390 pM, 1.56 nM, 25 nM, 100 nM), compared to the control analyte IL4 detected at the same concentration levels.

**Figure 8:**
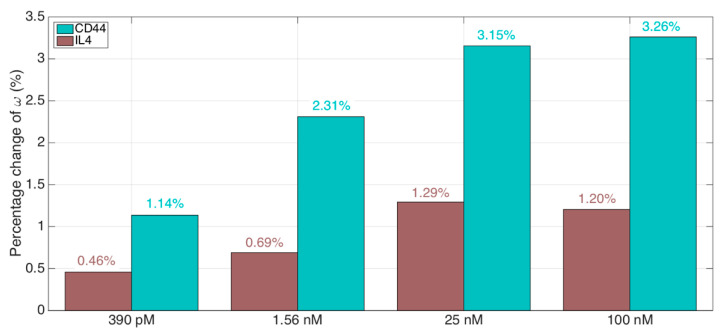
Evaluation of the specificity of the CD44 biosensor, using the KLT interrogation method. The chart shows the percentage change of the KLT output *ω*, calculated from the reference condition (6 pM, lowest concentration), for 4 different concentrations (390 pM, 1.56 nM, 25 nM, 100 nM) of the CD44 protein analyte compared to a control (IL4).

The results of the specificity analysis are shown in Figure 8, where we compare the change of KLT output *ω* from the reference condition (6 pM) observed for both CD44 and control IL4. We observe that the response to control is approximately 2.5 times inferior to the response to CD44, which validates the concept of the sensor. The specificity, observed between 390 pM and 100 nM, ranges from 30% to 41%, computed as the response to control normalized by the response to the CD44 analyte. This value is in analogy with other CD44 biosensors with lower signal (impedance decrease rate and current change, respectively) for control proteins [31,32], but these sensors were capable of working at lower concentrations.

### 4.4. Comparison between KLT and Feature Extraction

As a key validation of the KLT capabilities, we compared its performance to the spectral feature extraction and analysis method that has been proposed in previous works [20] for ball resonator demodulation. This benchmark assessed the quality of the spectral detection by comparing methods providing different outputs, by normalizing the responses in calibration with a known response.

For the KLT, as previously reported, we evaluated the change of the main normalized eigenvalue *ω*, computed by processing the reflection spectrum acquired through the OBR. For the feature extraction (FE) method, similarly to [20] we scanned the same reflection spectrum (filtered with the same low-pass digital filter) and measured the reflectivity change of the most significant feature. As observed in [20], the analysis of the intensity change tended to return a better accuracy (i.e., better R^2^) than the measurement of wavelength shift, when the detector was based on an accurate power meter as the OBR.

Since both responses are based on distinctive methods and different measurement units, we normalized their responses between 0 and 1, using a simple normalization as in the formula below:

(12) $$Y=\frac{y-y_{min}}{y_{max}-y_{min}}$$

where *Y* is the normalized response, *y* is the output of each demodulation method (KLT and feature extraction), and *y_max_*, *y_min_* are the maximum and minimum of the responses for each method (*ω* for then KLT, the spectral intensity for the FE). This normalization allows us to compare the different methods in the same framework.

The calibration scenario was the same as presented for RI calibration; this was a good choice because, as shown previously for several fiber-optic biosensors [2], for a small RI change (below 10^−2^ RIU) we can perform a small-signal analysis in which we can approximate that the sensor response changes linearly with the RI. As such, our analysis consisted of 10 data points of RI between 1.3478 to 1.3539.

In order to evaluate the quality of the detection, we performed a linear fit between the RI change and the normalized response *Y*. As a figure of merit, we consider the coefficient of determination R^2^ between the datapoints and the fitted data; a high R^2^ (>0.95) ensures that the method correctly demodulates the sensor, while a low value of R^2^ shows that datapoints are too scattered and therefore the demodulation method fails to correctly identify spectral changes.

**Figure 9:**
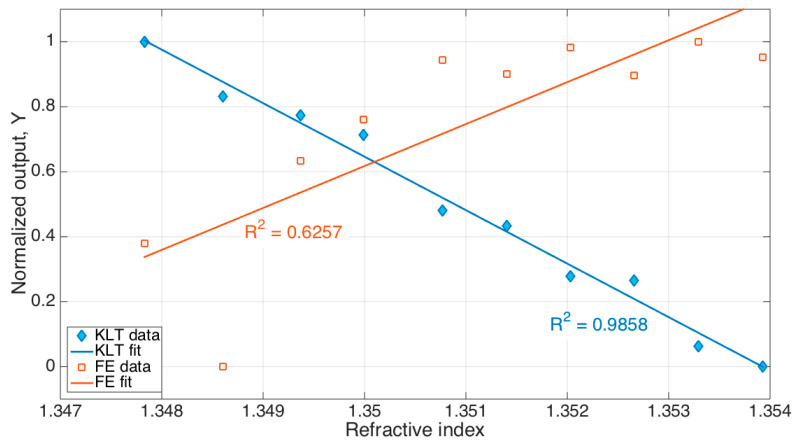
Comparison between KLT and feature extraction (FE) methods for demodulation of the ball resonator. The chart shows the normalized output (*Y*) as a function of the RI, obtained for each method. Datapoints have been interpolated with a linear fit; the R^2^ coefficient of determination shows the quality of the agreement between measured data and the fit.

The comparison between KLT and FE, presented for the previously described resonator, is shown in Figure 9. We see that while the KLT correctly identified the RI change with a reliable method, returning an R^2^ coefficient of 0.9858, the standard FE method achieved only an R^2^ value of 0.6257, hence failing to correctly demodulate the signal. The main reason is that while the KLT isolates the main eigenvector of the spectrum, which encodes the spectral change, the shallow spectral peaks overlapped with noise prevent the FE from achieving a clean detection, which results in poor calibration quality.

We can further extend the comparison between KLT and FE methods, in order to provide a more encompassing view of the capabilities of the KLT compared with the standard feature extraction and analysis. For this task, we fabricated five additional ball resonators, using the same method described in Section 2.1, and having a diameter ranging from 290 μm to 532 μm (respectively: 290, 466, 515, 521, 532 μm). Reflection spectra were acquired and measured using the same method described in Section 2.2, and further processed using the KLT and the FE methods, and each output was normalized in order to provide a comparison between the methods.

**Figure 10:**
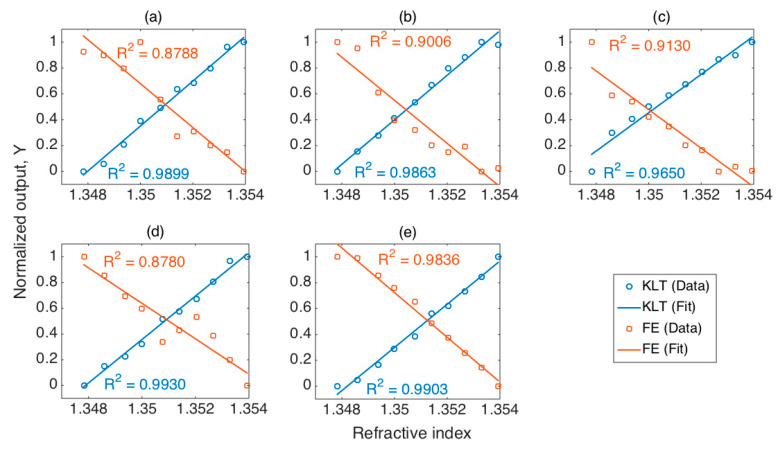
Comparison between KLT and feature extraction, for 5 different resonators. The charts report the normalized output *Y* for each method, as a function of refractive index (10 RI datapoints from 1.3478 to 1.3539). All ball resonators were fabricated using the same method based on CO_2_ laser splicer, and had different diameters: (**a**) 290 μm; (**b**) 466 μm; (**c**) 515 μm; (**d**) 521 μm; (**e**) 532 μm.

We can observe, from the view in Figure 10, that in all the cases, the KLT was able to process the spectral fingerprints of the ball resonators with success, returning an R^2^ coefficient ranging between 0.965 and 0.993; conversely, the FE method performed a correct (R^2^ > 0.95) identification of the spectral feature only in one case (R^2^ = 0.984), while for all the other four cases the coefficient of determination ranged between 0.879 and 0.913, below the threshold for correct identification. Of all the five ball resonators used in this study, the KLT method returned a better R^2^ than the FE method, at no computational expense, since, in both cases, the computation time is instantaneous (<10 ms).

Overall, this benchmark shows that the KLT adapts the output to each spectral signature, without inputting a priori information into the detection; on the other hand, the FE method works well only in ball resonators that exhibit a clear spectral feature, which leads to a much inferior rate of successful detection.

## 5. Discussion

Optical fiber ball resonators are a powerful tool for refractive index sensing [20] and biosensing [21], as they combine a single-mode fiber-optic structure, which allows the use of telecom-grade interrogators with high precision and wavelength sensitivity, a high sensitivity rating comparable with plasmonic grating sensors [13] and U-bent fiber biosensors [33], and a splicing recipe that can be fabricated in large volume. In particular, the manufacturing process requires a very short fabrication time, of just a few seconds duration, reusing a method developed for the fabrication of diffractive lenses with fibers having a larger core [34].

In this scenario, the KLT plays a significant role because it deals with the main weakness of ball resonators used for RI sensing and biosensing, which is the quasi-random pattern of the spectral signature of each ball resonator, which also appears to be not significantly related to geometrical parameters, such as size and eccentricity.

In particular, the KLT operates as a “black box”, taking as input the reflection spectra and returning an output that is neither an intensity change nor a wavelength shift, but it directly links to the change of intensity and wavelength of the spectrum. While refractometers and biosensors express their performance figures in terms of sensitivity to wavelength shift (usually expressed in nm/RIU) or intensity change (expressed in dB/RIU), the KLT applied to the ball resonators reports a sensitivity rating in terms of variation of *ω* as a function of the RI, which can be expressed as RIU^−1^ or in %/RIU. Nevertheless, since the quality of the detection is the most important factor in the demodulation of the ball resonator, we can observe that the KLT achieves a performance rating superior to the spectral feature extraction and analysis. In particular, when detecting the RI in a small range, the KLT has always succeeded in detecting the spectrum of ball resonators having a reflectivity level sufficient to perform a detection (return loss > −70 dB), while the FE achieves a successful detection in only a few resonators (about one successful detection for every six devices fabricated with sufficient reflectivity).

The KLT method finds important applications in signal processing, as it is a versatile method for analyzing complex signals. This method was originally developed in order to extract very weak signals from noise, using the bordered autocorrelation method [28] that allows the complexity to be reduced into incremental contributions. This method deals with signals having a large sample size; however, the KLT has recently found applications in coarsely sampled signals [35], exploiting its capability to extract signals with lower noise contribution but having low sample sizes. Table 1 reviews some of the applications of KLT, in its original implementation and more recently in fiber optic sensing.

This work proposes to convert the KLT from the analysis of long signals, which is a high-computational complexity problem, into a low complexity problem that can be solved in few milliseconds by using a downsampling routine. We expect that, through analyzing the main eigenvalue that constitutes the most important feature of the spectrum, we can even further increase the performance, particularly when looking at interrogating ball resonators with analyzers with a lower cost and inferior resolution bandwidth. In terms of application, the key benefit of the KLT, besides improving the detection, is that the user should no longer search for the most significant features, which might also change when the medium surrounding the sensor varies, but the KLT automatically identifies the most significant feature; then, by considering only the main eigenvalue, this is singled out from the surrounding noise without having to apply any prior knowledge of the sensor spectra.

The availability of a powerful detection method, such as the KLT, is an important asset for interrogating sensors fabricated with CO_2_ laser splicers [38,39], which yield rapid and repeatable fabrication but with spectral characteristics that are often hard to process with an FE method.

## 6. Conclusions

In conclusion, we presented a method based on the Karhunen−Loeve transform for the interrogation of optical fiber ball resonators used in refractive sensing and biosensing. Optical fiber ball resonators have the advantage of a single-step rapid and reliable fabrication process, which can make a significant step up in terms of volume manufacturing. The main drawback, however, is that the spectrum has a quasi-random pattern, which significantly hampers the detection method.

The undersampled KLT method presented in this paper shows great effectiveness in the demodulation of the reflection spectra of the ball resonator, outperforming the feature extraction methods used so far [20]. In particular, its main strength is that the eigendecomposition method at its core can self-adapt to any spectrum, which fits very well the almost random nature of the spectral fingerprints observed in the ball resonators. By optimizing the downsampling rate, the KLT method is both accurate and computationally fast.

We presented the effectiveness of the KLT and its application to RI sensing, achieving a sensitivity rate of 1593.6 RIU^−1^; subsequently, we validated its application to biosensing by functionalizing one ball resonator to CD44 biomarker detection, achieving a 19.7 pM limit of detection and a ~40% specificity in the worst-case scenario.

## Figures and Tables

**Table 1:** Review of some recent implementations of KLT in signal processing related to sensing data and analysis, and practical applications.

Reference	KLT Implementation	Application	KLT Output
Maccone 2010 [24]	KLT—Bounded autocorrelation matrix	Extract a weak narrowband tone buried in white noise	Extraction of the principal eigenfunction
Maccone 2006 [29]	KLT of Brownian motion	Optimization of special relativity computations	Rescaled-time eigenfunctions
Lee 1999 [36]	KLT preprocessing of discrete cosine transform	Multispectral image compression	Principal component analysis
Sharma 2018 [37]	KLT fused to discrete wavelet transform	Cryptography: hiding imperceptible data in images	Eigenfunctions of the SVD
Tosi 2015 [35]	KLT-SVD of coarsely sampled signals	Interrogation of fiber Bragg grating sensors	Principal eigenvalue
This work	Undersampled KLT	Interrogation of optical fiber sensors having quasi-random spectrum	Normalized principal eigenvalue

## Data Availability

The data presented in this work are not available to the public, but can be obtained from the authors upon reasonable request.

## Supplementary Materials

The following are available online at https://www.mdpi.com/article/10.3390/s21206721/s1, Table S1: The fabrication parameters of the ball resonator using CO_2_ laser splicer (Fujikura LZM-100).
