# Supplementary material for: Optical Fiber Ball Resonator Sensor Spectral Interrogation through Undersampled KLT: Application to Refractive Index Sensing and Cancer Biomarker Biosensing
Source: Sensors (Basel). 2021 Oct 10;21(20):6721. doi: 10.3390/s21206721 (PMC8537289; doi:10.3390/s21206721)
Supplement: Supplementary file 1 [file sensors-21-06721-s001.zip › sensors-1400201-supplementary.pdf]

Supplementary material

Optical fiber ball resonator sensor spectral interrogation through undersampled KLT: application to refractive index sensing and cancer biomarker biosensing

Daniele Tosi [1,2,*], Zhannat Ashikbayeva [1], Aliya Bekmurzayeva [1,2], Zhuldyz Myrkhiyeva [1], Aida Rakhimbekova [1], Takhmina Ayupova[1], and Madina Shaimerdenova [1]

[1]Nazarbayev University, School of Engineering and Digital Sciences, 010000 Nur-Sultan, Kazakhstan
[2]National Laboratory Astana, Laboratory of Biosensors and Bioinstruments, 010000 Nur-Sultan, Kazakhstan
*Correspondence: daniele.tosi@nu.edu.kz;

Table S1. The fabrication parameters of the ball resonator using CO_2 laser splicer (Fujikura LZM-100). Pre-heat value and the power levels are reported in bit units, as displayed on the splicer.

Parameters	Diameters (*x*, *y* axes), µm
	**490-484**
Pre-heat (bit)	1
Absolute power (bit)	342
Relative power (bit)	90
Break-add power (bit)	100
Feeding speed (mm/sec)	0.2
Rotator speed (deg./sec)	180
Diameter adjustment (µm)	-10
